# Integrated Dentist–Nurse Collaborative Care Model for Caries Management in China

**DOI:** 10.3390/dj14070430

**Published:** 2026-07-12

**Authors:** Jiaohong Liu, Haohao Wang, May Lei Mei, Chloe Meng Jiang, Lei Cheng, Ling Zhang

**Affiliations:** 1 State Key Laboratory of Oral Diseases & National Center for Stomatology & National Clinical Research Center for Oral Diseases, West China Hospital of Stomatology, Sichuan University, Chengdu 610041, China; liujiaohong@scu.edu.cn (J.L.); wanghaohao@scu.edu.cn (H.W.); 2 Department of Cariology and Endodontics, West China Hospital of Stomatology, Sichuan University, Chengdu 610041, China; 3 Sir John Walsh Research Institute, Faculty of Dentistry, University of Otago, Dunedin 9016, New Zealand; 4 Faculty of Dentistry, The University of Hong Kong, Hong Kong 999077, China

**Keywords:** long-term nursing, caries management, Dentist–Nurse Collaboration

## Abstract

Dental caries, as a common chronic disease with high global prevalence, has undergone a paradigm shift from a traditional treatment-oriented model to a management approach focused on controlling caries risk factors while implementing personalized preventive and therapeutic measures. Confronted with limited medical resources and the additional workload imposed by long-term caries management, the Integrated Dentist–Nurse Collaborative Care Model emerges as a potentially effective pathway to address these challenges. Through task-shifting and collaboration, this model leverages the pivotal role of dental nurses in prevention, assessment, health education, and follow-up management, which may enhance the efficiency of healthcare services. This article elaborates the approaches of implementing the Dentist–Nurse Collaborative Care Model in caries management in China. This includes dental nurse-led health education and assessment conducted before and after treatment, where behavioral guidance is utilized to enhance patients’ self-management capabilities. During the clinical stage, dentists and dental nurses collaborate to advance both caries risk management and caries lesion management, formulating personalized plans based on caries risk assessment and treatment difficulty assessment. In the continuity of care phase, through long-term follow-up and plan adjustments, dentists and dental nurses work together to maintain patients’ oral health, ultimately achieving a fundamental transition from single-episode treatment to comprehensive long-term caries management.

## 1. Introduction

Dental caries is a chronic, progressive, and destructive disease that occurs in the tooth hard tissue under the influence of multiple factors, predominantly bacteria. This disease is widespread globally and has been listed as one of the three major non-communicable diseases globally by the World Health Organization [1]. Statistics from The Lancet on 354 major global diseases reveal that permanent tooth caries ranks second in incidence, while deciduous tooth caries ranks fifth [2]. The fourth oral health epidemiological survey conducted in China also revealed that the prevalence of caries among different age groups ranged from 41.9% to 98% [3]. Those reports indicate that dental caries is not merely a “localized oral disease” as traditionally perceived, but rather a “major global public health challenge”. It is imperative to incorporate caries prevention and control into the chronic disease management system and to adopt a new interdisciplinary prevention and treatment model. Therefore, the concept of caries management was introduced into modern dentistry and is now well-recognized. Its aim is to shift from the traditional treatment-oriented model to a caries management model that controls the progression of caries by controlling the risk factors of caries and implementing personalized prevention and treatment measures [4,5]. Furthermore, a comprehensive caries management plan covering the whole life cycle has also been proposed, which includes group management of caries for patients of the same age group based on their physiological characteristics, as well as individual management of caries at different risk levels [6]. The importance of caries management lies not only in reducing the prevalence rate and medical costs, but also in improving the long-term oral health and quality of life of patients. Efficient and continuous management has become a core task of the oral public health system [7]. Confronted with limited medical resources and the additional workload imposed by caries management, dental team collaboration emerges as a potentially effective pathway to address these challenges. To explore the feasibility of dentist–nurse collaboration in the context of caries management, we conducted a literature search to present existing evidence on collaborative care models, task-shifting strategies, and the expanded roles of dental nurses in oral healthcare, with particular emphasis on the practical workflow of the collaborative model in applications in China. The search strategy and selection process are described below.

## 2. Literature Search Strategy

A comprehensive review was conducted using PubMed and Web of Science. Most of the searches were performed from 2016 to the present. Only peer-reviewed articles published in English were considered. Boolean operators were applied to combine keywords: TS=(((“dental caries” OR “tooth decay” OR caries) AND (“interprofessional collaboration” OR “nurse-physician collaboration” OR “doctor-nurse collaboration” OR “multidisciplinary care” OR “shared care” OR “team-based care” OR “nurse-led” OR “physician-nurse relations”)) AND (“oral health” OR “dental care” OR “integrated care” OR “oral health management”)). Titles, abstracts, and full texts were screened for relevance and rigor. All retrieved records were exported to EndNote 21. Duplicates were identified and removed, first automatically by the software and then manually by two independent reviewers.

## 3. Caries Management of Integrated Dentist–Nurse Collaborative Care Model in China

With the growing adoption of comprehensive caries management, approaches to dealing with dental caries are undergoing a significant shift, where caries control no longer only focuses on treatments themselves, but also includes management procedures such as assessing and controlling the risk factors to reduce the incidence rate, providing appropriate treatment for existed caries, conducting regular follow-ups and restoration maintenance after treatment, etc. A central aspect is the concept of teamwork, where every member of the oral healthcare team is involved [8]. When implementing caries management in daily visits and oral health education, this will typically increase the workload. Therefore, it is advisable to adjust the team collaboration and arrangements accordingly [4]. In most countries, dentists work alongside dental ancillary personnel to diagnose and treat diseases. Within China’s dental healthcare system, dental ancillary personnel refer to dental nurses—registered nursing professionals who have undergone specialized training. Their responsibilities encompass a broad scope, including clinic and material management, clinical assistance such as implementing the four-handed dentistry technique, patient education, and a range of other tasks that support dentists in their work. With their support, dentists can focus primarily on decision-making processes, such as treatment planning and execution, while routine and ongoing management tasks such as health education and long-term follow-up can be led by the dental nurse team. The Integrated Dentist–Nurse Collaborative Care Model may make the prevention, treatment, and management of caries more effective and may also influence patients’ behavior in the long run.

## 4. Extended Roles of Dental Nurses in Caries Management

In the concept of dentist–-nurse integration, the deep evolution and empowerment of dental nurses may represent a key opportunity for enhancing the efficiency of dental disease management [9]. Dental nurses need to evolve from their traditional supportive roles to become core participants in the prevention, treatment, and continuous management of dental caries, taking on functions such as risk assessment, health education, behavioral intervention, and long-term follow-up [10], which has the potential to fill the gap in preventive, educational, and continuous care that the traditional model fails to cover, thus improving the effectiveness of caries management [11].

### 4.1. The Role of Dental Ancillary Personnel in Different Countries

The dental ancillary personnel have been given various names based on their technical characteristics. In the United States, they are called dental assistants and dental hygienists [12,13]. “Dental assistants” mainly assist at the dental chair, handle instrument transfers, control infections, and perform four-handed operations [12]. “Dental hygienists” independently carry out scaling, preventive treatments, and home care tips [13]. Their training and certification systems are separated, and their responsibilities are clearly defined. In New Zealand, dental nurses are divided into enrolled nurses and registered nurses. Enrolled nurses primarily provide foundational support, such as assisting patients with daily activities, monitoring vital signs, and administering routine medications. Registered nurses, by contrast, take on more complex tasks, including conducting comprehensive health assessments, developing personalized care plans, and collaborating with doctors to implement treatments. Their role also extends to health education and community outreach, making them key contributors to both individual patient recovery and public health promotion [14]. In Hong Kong, they are called dental nurses. They not only perform routine chair-side tasks such as passing instruments, managing patient records, and maintaining a sterile clinical environment, but also diagnose minor conditions, prescribe certain medications, and lead specialized care teams to address complex medical cases [15]. In the UK, the dental auxiliary system features a clear division of responsibilities: dental nurses mainly provide chairside support, infection control, and patient reassurance, along with other assisting tasks, while dental hygienists focus on more independent clinical procedures, such as preventive treatment, periodontal care, and oral health education [16]. In Japan, they are called dental hygienists [17], and their role focuses more on disease prevention, daily management, and health education, which is distinct from the functions of treatment assistants. In China, dental nurses, who perform four-handed operations, are responsible for infection control and carry out patient health education, etc.

By comparing the functional differences among dental support teams across different countries, it appears that the role of dental nurses in China has fused the core functions of multiple roles in the international system, as shown in Table 1. However, during the diagnosis and treatment process, the doctor and the nurse have clear primary and secondary divisions of labor, with dental nurses mainly performing assisting tasks and having relatively limited independent decision-making authority [18]. In contrast, in many western countries, the nursing team in dental settings is organized into more distinct and specialized roles (such as dental hygienists and dental assistants). These professionals undergo separate training and certification programs and are legally permitted to perform a broader range of independent or semi-independent duties—including scaling, preventive fluoride application, patient education, and even certain diagnostic assessments—within their defined scope of practice. However, overall, both China and the international community have adopted a model in which dentists work alongside dental supporting teams to carry out clinical care. Therefore, the Integrated Dentist–Nurse Collaborative Care Model in China discussed in our article may also offer valuable insights and serve as a reference for caries management approaches in other countries.

### 4.2. Dental Nurses May Align Closely with the Requirements of Caries Management

With the continuous development of stomatology, modern caries management has shifted from “treatment-oriented” to “long-term management combining prevention and treatment”. The successful implementation of this approach may depend on the coordination of three core elements: timely and accurate risk assessment, lifelong behavioral intervention, and caries management for special populations. In this process, the active involvement of the dental nursing force is of importance [31].

#### 4.2.1. Dental Nurses Show Advantages in Risk-Based Caries Management

The breakthrough in modern caries management lies in the caries risk assessments, which involves identifying high-risk groups for caries and then taking corresponding preventive or therapeutic measures to regulate various risk factors that affect the occurrence and development of caries, thereby controlling the progression of caries and restoring the structure and function of teeth [32]. Involving dental nurses in caries management may enhance both the scope and timeliness of care. After systematic training, dental nurses may conduct multi-angle and multi-level risk assessments (assessing buffering capacity and flow rate of saliva, quantifying the concentration of cariogenic bacteria using test strips, and tracking sugar exposure frequency through patients’ diet diaries, etc.), facilitating a broader review of patients’ caries risk factors [33]. Furthermore, it may contribute to earlier intervention. Dental nurses could perform a preliminary oral risk assessment for each patient via online questionnaires and establish a dental alert record. They might also deliver early-risk control education through online channels such as public accounts. When dentists are still limited by clinic time and professional division of labor, dental nurses—owing to their more frequent contact advantages—are expected to potentially advance the risk identification node [34].

#### 4.2.2. Dental Nurses Are Suited for Behavior Intervention Throughout the Entire Life Cycle

Another major element of caries management is behavioral intervention throughout the entire life cycle, with strategies tailored to the physiological characteristics of different age groups for targeted, group-based disease control [6]. In the field of long-term caries management throughout the entire life cycle, its essence of “education as the main approach, with long-term follow-up” appears to correspond with the professional role of dental nurses [6]. In terms of health education, dental nurses possess inherent communication advantages. They can adopt a non-authoritative and equal approach to transform complex medical principles into relatable language (for example, using the story of “the tooth plaque monster” to guide children to brush their teeth). This empathetic patient education model can increase the acceptance rate of patients [35]. In terms of long-term tracking, the continuity care ability of dental nurses constitutes a distinctive advantage. By establishing oral health follow-up archives spanning several years or even decades, dental nurses can connect discrete treatment events into a coherent health journey. Based on long-term care and continuity care data, dental nurses may more sensitively detect risk evolution nodes, ultimately advancing the prevention threshold of caries in the time dimension. Yoon et al. investigated the oral health status of residents in long-term care institutions in four provinces of Canada and found that 57.6% of the residents receiving long-term oral care had intact teeth, with an average of 16.4 teeth, and their oral conditions were significantly better than those of residents who did not receive long-term care (32.7% had intact teeth, with an average of 13.4 teeth) [36].

#### 4.2.3. Dental Nurses Are Qualified for the Adaptive Caries Management for Special Populations

Special populations (such as those with dementia, disability, or post-stroke sequelae who have severely limited self-care abilities) often encounter difficulties in understanding instructions and having restricted physical movements, making it challenging to safely and effectively implement the standard caries management process. How to efficiently manage caries for such special patients has always been a key difficulty and bottleneck [37]. Dental nurses possess professional patient education skills and nursing techniques, and through repeated contact, they may reduce the psychological defenses of special groups, contributing to a sense of security, and make special groups more likely to transform from medical-resistant individuals to active participants. Based on the oral examination results of thousands of international athletes participating in the Special Olympics, Waldman et al. proposed an oral health screening form for patients with intellectual and developmental disorders and emphasized that dental nurses play an important role in providing oral examinations, preventive services, and referrals to dental medical institutions for special patients [38].

### 4.3. Functions Performed by Dental Nurses in Caries Management

In contemporary caries management, dental nurses are expected to transcend their traditional auxiliary roles and adopt an integrated professional identity as health promoters, operators, educators, and researchers. This composite role positions them as a link between medical technology and humanistic care, which may facilitate the transition of caries management from fragmented interventions toward systematic and continuous approaches.

As health promoters, dental nurses should undertake a central role of risk monitoring. By relying on systematic caries risk assessments and standardized detection procedures for active caries, dental nurses may identify potential risk factors and active carious diseases and establish dynamic early warning files [39]. On this basis, after undergoing professional training, dental nurses may independently implement primary intervention measures, such as conducting fluoride application procedures and performing pit and fissure sealants [40].

In their role as operators, dental nurses should demonstrate the professional value of precise coordination during clinical procedures. By anticipating the treatment process, accurately transferring the instruments, and continuously maintaining a clear surgical field, the nurse could improve the treatment efficiency and operational safety. The technical value of dental nurses lies not only in the optimization of time management, but also in creating a stable treatment environment: adjusting the body position to reduce muscle tension of the patient, using aspiration system to maintain the operating field, and using language to soothe and alleviate the treatment anxiety. This seamless collaboration enables the dentist to focus on the implementation of core techniques, jointly ensuring the accuracy and comfort of the treatment process [41].

As educators, dental nurses master professional health communication skills and behavior change techniques. Through various publicity channels, they may help patients gain a deeper understanding of caries risk factors and develop a more accurate perception of disease severity and personal susceptibility. This could enhance the oral health literacy and practical skills of both patients and the public [42].

As researchers, dental nurses may conduct research on evidence-based practice and nursing innovations in dentistry, exploring nursing strategies for specific populations and conducting research on specific clinical issues. Through these efforts, caries management may be kept up to date with emerging evidence and continuously enriched with novel scientific perspectives [43].

**Figure 1:**
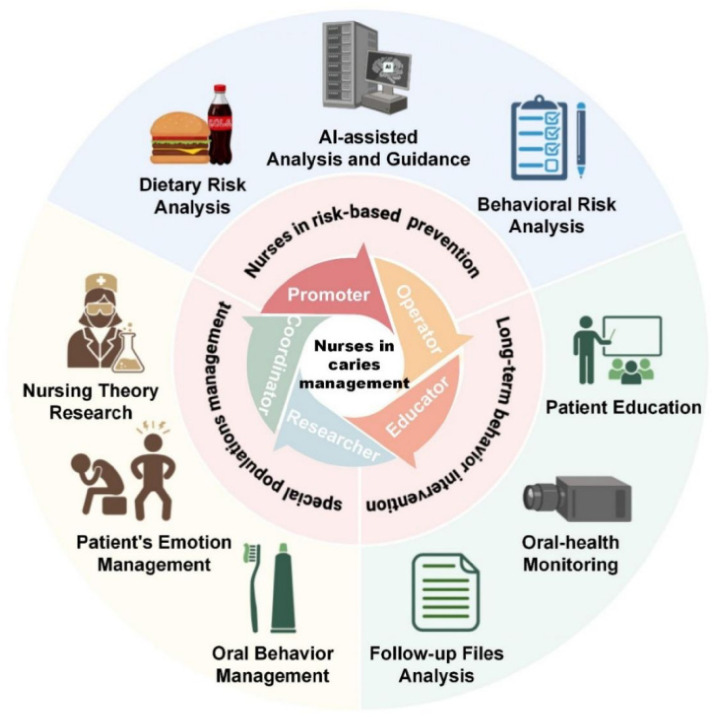
The role of dental nurses in caries management.

Health promoters perform real-time risk assessments, operators optimize treatment efficiency, educators improve patients’ oral healthcare skills, and researchers drive system evolution. This dynamic integrated professional identity enables dental nurses to break through traditional medical boundaries and plays important roles, making the dentist–nurse integration in caries management become possible, as shown in Figure 1.

## 5. Approach of Integrated Dentist–Nurse Collaborative Care Model for Caries Management in China

As mentioned above, the greater involvement of dental nurses is of importance in caries management. On this basis, the Integrated Dentist–Nurse Collaborative Care Model for caries management may break the traditional medical division barriers, achieving a deep integration of knowledge, skills, and responsibilities between dentists and dental nurses [44].

### 5.1. The Manifestation of Dentist–Nurse Collaboration for Caries Management

#### 5.1.1. Integration of Goals and Concepts: All Efforts Aimed at Caries Prevention and Control

By breaking down the functional barriers between dentists and the nursing team, dentists and dental nurses are no longer confined to their respective professional goals (for example, dentists focus on disease treatment while dental nurses focus on basic nursing tasks) but jointly take responsibility for the long-term oral health outcomes of patients.

#### 5.1.2. Deep Integration of Work Processes: Seamless Connection and Efficient Collaboration

Prevention (education, behavioral intervention) and treatment (clinic intervention, clinic treatment) are carried out simultaneously to achieve lifelong caries management. Information sharing and real-time communication are realized through electronic health records (EHR), management apps, etc.

#### 5.1.3. Complementary Extension of Roles and Abilities: Maximizing Professional Value

**Figure 2:**
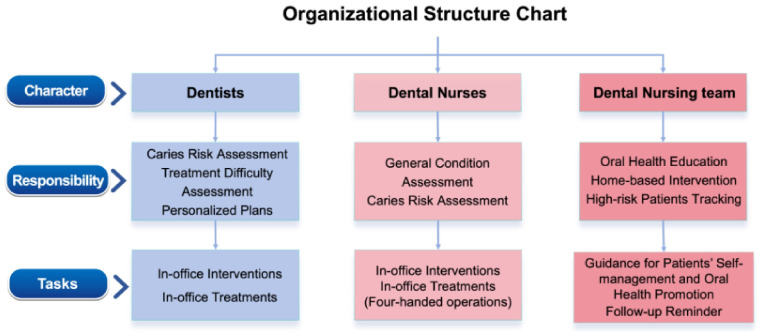
Organizational structure chart of Integrated Dentist–Nurse Collaborative Care Model for caries management in China.

Dental nurses are encouraged to transform from passive executors to a “health promoter”, “operator”, “educator”, and “researcher”—a four-in-one professional identity. They may undertake the “front end” (caries risk assessment, prevention) and “back end” (follow-up, maintenance) in caries management, becoming the main health advisor for patients. Dentists who previously performed extensive repetitive caries management tasks can then be relieved from work, allowing them to devote more energy to diagnosing and treating complex cases, making critical clinical decisions, overseeing quality control, and engaging in technological innovation. The organizational structure chart of the Integrated Dentist–Nurse Collaborative Care Model for Caries Management in China is shown in Figure 2.

### 5.2. Integrated Dentist–Nurse Collaboration Process

**Figure 3:**
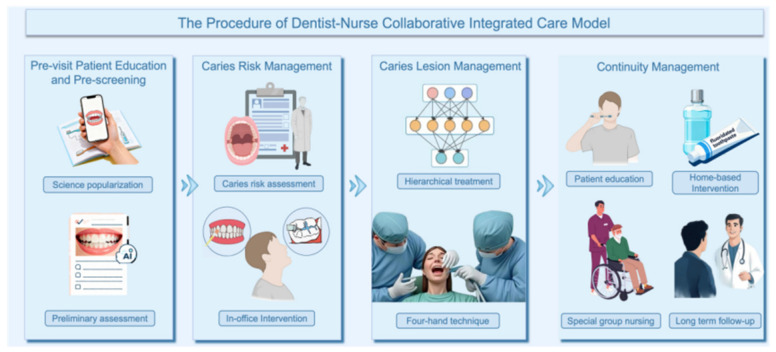
The procedure of Integrated Dentist–Nurse Collaborative Care Model for caries management in China.

The procedure of the Integrated Dentist–Nurse Collaborative Care Model in China is illustrated in Figure 3. It comprises four key components: pre-visit patient education and pre-screening before the patient’s arrival; an integrated approach to caries risk management and caries lesion management during the in-clinic visit; and continuity management following treatment.

#### 5.2.1. Pre-Visit Patient Education and Pre-Screening

This segment is aimed at implementing primary prevention measures, enhancing public awareness of oral health, conducting targeted education for key groups, and identifying high-risk individuals at an early stage. It is primarily spearheaded by dental nurses, who engage in direct communication with patients to deliver educational content and conduct pre-consultation screenings. Dentists play a supportive role by verifying the scientific accuracy of health education materials and providing professional training to dental nurses. Other healthcare professionals, such as community doctors, health educators, and dental hygienists, can also be incorporated to expand the outreach impact.

1. Diversified Science Popularization Platforms

Online health education is conducted through the hospital’s official social media accounts. Content may cover accessible topics such as proper brushing techniques and dietary advice, with public engagement facilitated through interactive formats like Q&A sessions [45]. Offline initiatives may include health lectures and free screenings organized in collaboration with communities, schools, enterprises, and nursing homes. Interactive experience zones can also be set up in public venues such as shopping malls and libraries. To address diverse population needs, health education materials can be tailored accordingly: animations, picture books, and interactive brushing apps can be designed for children, while large-print or audio-based formats can be developed for older adults. Simplified oral care manuals can also be created for family members caring for individuals with dementia [46].

2. Tailored health promotion for distinct age groups and vulnerable populations

The dental team can develop science-based educational content tailored to distinct age groups and distribute it precisely to target populations by placing materials in relevant hospital departments through reception handbooks, corridor posters, television broadcasts, and other channels. This could help to achieve community-based caries management covering the whole life cycle [6]. For example, in the pediatric dentistry department, health education such as parental guidance (feeding practices, snack control) and behavior management (treatment cooperation) and gamified oral care tools can be provided for public awareness [46]. In the obstetrics and gynecology department, knowledge about pre-pregnancy oral examination and oral health maintenance during pregnancy can be disseminated. In the geriatric dentistry department, the elderly can be helped to recognize root caries prevention, denture-related caries, as well as oral diseases related to systemic diseases and medications. In the rehabilitation department, we can focus our efforts on special groups such as people with dementia or disabilities and help them and their families understand simplified oral care plans, such as electric toothbrushes and water flossers. In the oncology-related departments or radiotherapy clinics, some educational content about pre-therapy oral assessments and post-therapy oral maintenance can be provided. Furthermore, these contents can be expanded through collaborations with kindergartens, schools, communities, nursing homes, and other institutions.

3. Preliminary Assessment

AI-assisted preliminary assessments are implemented to identify individuals at high risk of dental caries by analyzing simple questionnaires and intraoral photos [47]. Notably, before using AI tools for dental caries assessment or remote monitoring, the consent of the patient should be obtained, and technical measures such as data encryption, access control, and secure storage should be adopted to ensure the confidentiality and security of the patient’s images and personal health information. Moreover, the output results of the artificial intelligence should only serve as a support tool for clinical decision-making, rather than an independent diagnostic basis. Based on the assessment results, electronic health records may be established to facilitate information for dentists. This process helps direct identified high-risk individuals to seek timely clinical consultation or enroll in structured prevention programs [48]. To extend the reach of oral health initiatives, community resources can further be integrated through collaboration with social workers, schoolteachers, and aged care personnel, enabling the consistent implementation of daily oral care and protective measures in community settings.

#### 5.2.2. In-Clinic Care: Parallel Implementation of Caries Risk Management and Caries Lesion Management

**Figure 4:**
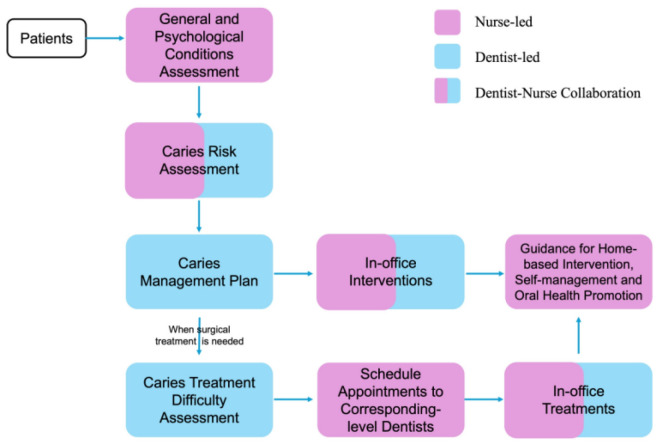
In-office workflow of Integrated Dentist–Nurse Collaborative Care Model for caries management in China.

Caries management comprises two key components: caries risk management and caries lesion management. Caries risk management focuses on controlling caries by reducing the caries risk factors, enhancing protective factors, and assisting with patient behavior modification to improve individual susceptibility to caries [49]. This prevention-oriented approach spans the entire caries management process. The commonly used caries risk assessments include the ADA caries risk assessment [50], American Academy of Pediatric Dentistry caries risk assessment (AAPD) [51], caries management by risk assessment (CAMBRA) [4] and Cariogram [52]. Caries lesion management refers to the development of personalized treatment plans based on the patient’s caries risk assessment and caries treatment difficulty assessment once cavitation has occurred. This treatment-centered approach entails referrals to general practitioners, cariology specialists, or clinical experts based on the assessed treatment difficulty level, followed by conducting treatment, periodic follow-ups, and long-term restorative maintenance to ultimately achieve the goal of controlling caries progression and restoring tooth function. The treatment difficulty assessment system used in caries lesion management was firstly proposed by West China Hospital of Stomatology (WCHS) in 2022 [5]. This article will take CAMBRA caries risk assessment and WCHS treatment difficulty assessment as examples to demonstrate the clinical application workflow of caries management in China, as shown in Figure 4. This workflow has undergone clinical pilot implementation in our hospital. Preliminary findings suggest that it sustains procedural continuity and collaborative efficiency in caries management. While quantitative efficacy data from large samples are not yet available, the observed smooth interprofessional collaboration, patient acceptance, and information system support have already provided initial evidence for its clinical feasibility.

1. Caries risk management approach

Caries risk management requires collaborative involvement and coordinated efforts between dental nurses and dentists. During patient visits, the nurse initiates the process by conducting a comprehensive assessment and communication. This begins with a systemic evaluation, including the measurement of vital signs (such as blood pressure and heart rate) and detailed inquiry and documentation of the following: systemic disease history (particularly cardiovascular diseases, diabetes, Sjögren’s syndrome, and history of radiotherapy or chemotherapy), pregnancy status, medication history, allergy history, past dental treatments, and lifestyle habits (such as tobacco and alcohol use, and specific dietary patterns), as well as the identification of conditions requiring special attention (e.g., the need for prophylactic antibiotics in patients with valvular heart disease), with any abnormalities promptly reported to the dentist. Subsequently, the nurse may then assist in initiating the preliminary components of the caries risk assessment of CAMBRA, including protective factors and biological or environmental risk factors. The dentist will then step in to integrate information and make clinical decisions. The dentist should first perform radiographic and clinical examinations and complete the clinical exam part of CAMBRA, scoring the patient’s caries risk level (low, moderate, high, or extreme). Based on the caries risk level, the dentist will then develop a personalized caries management plan, which includes in-office interventions such as fluoride application frequency, pit and fissure sealants and recall intervals, as well as home-based interventions like toothbrushing frequency, use of mouthwash, and dietary habit adjustments. If the patient needs in-office interventions, procedures such as fluoride application and sealants will be performed collaboratively by the dentist and nurse. In China, these interventions procedures are performed by the dentist with the nurse assisting. Finally, the dentist is responsible for completing postoperative instructions and medical record documentation, while home-based intervention guidance, patient self-management education, and oral health promotion can be handled by the nurse, who may utilize educational materials previously developed by the dental team for this purpose.

2. Caries lesion management approach

If the patient has been diagnosed with caries requiring surgical treatment, the following steps should be implemented. Firstly, the WCHS treatment difficulty assessment is used to comprehensively evaluate treatment difficulty through various factors including involved tooth surfaces and sites, depth of carious lesions, technical types, patients’ caries risk level, and other contributing factors like mouth opening, pharyngeal reflex, etc. Based on this evaluation, patients are classified by the dentist into treatment difficulty grades I, II, or III. Then patients are scheduled by nursing coordinators for appointments with corresponding-level practitioners (general dentists/specialists/experts) according to their assessed difficulty level. On the day of treatment, the nurse helps to verify the patient’s preoperative condition, such as performing chairside blood pressure measurements for hypertensive patients and confirming whether prophylactic antibiotics have been administered to those with valvular heart disease, to ensure the patient’s safety during the procedure, followed by treatment procedures involving collaboration between both dentists and dental nurses, as detailed in the next section regarding the treatment process for caries. After the treatment, the dentist is responsible for providing postoperative instructions and completing medical documentation, while the nurse conducts oral health education utilizing previously developed educational materials.

3. Treatment Process for Caries (Four-handed Technique)

During the treatment stage of dental caries, a graded referral mechanism based on the assessment of treatment difficulty is implemented, and the nursing team simultaneously construct a hierarchical configuration of capabilities [53]. Throughout the entire operation process, a standard four-handed operation procedure is carried out. Dental nurses are in the assistant area at the 2–4 o’clock or 9–12 o’clock position relative to the dentist, closely cooperating with each step of caries treatment such as debridement, cavity preparation, moisture isolation, filling, and polishing [54]. During the operation, the dental nurses precisely transfer instruments, mixed materials, and aspirate saliva to maintain a clear surgical field, while the dentist focuses on the treatment, which could shorten the treatment time and improve the treatment quality [55]. Additionally, throughout the process, dental nurses could provide real-time comfort and explanations to patients, helping to alleviate anxiety and enhance overall satisfaction.

#### 5.2.3. Continuity of Care: Long-Term Follow-Up and Plan Adaptation

In long-term follow-up care, dental nurses serve as the primary coordinators, taking charge of appointment reminders, performing basic examinations such as plaque disclosure and photographic documentation, and providing oral hygiene guidance. Dentists primarily make decisions regarding necessary new treatments, oversee restoration management, and adjust care plans based on changes in the patient’s caries risk status.

To be more specific, a personalized follow-up plan is generated based on the patient’s CAMBRA caries risk level. The preparation for the follow-up is primarily led by the nurse, who contacts the patient with an appointment reminder 1 to 2 weeks in advance and retrieves the patient’s records in advance, including their medical history and past treatment details. On the day of the follow-up, the nurse begins by performing basic examinations and evaluating patient behaviors, such as plaque disclosure recording, the patient’s toothbrushing behavior checking, dietary counseling, and reassessing the patient’s general health status. The dentist then joins the process, reassesses the patient’s caries risk level using the CAMBRA forms to determine whether it has decreased following in-office interventions and the patient’s self-management efforts, and adjusts the management plan according to the updated caries risk level. During this stage, the dentist conducts an oral examination and performs necessary radiographic checks to diagnose any new or recurrent caries requiring treatment. Existing restorations are also evaluated and maintained by the dentist based on modified USPHS criteria [56]. Subsequently, depending on the updated CAMBRA caries risk level, the patient receives corresponding in-office interventions, such as fluoride application. If new or recurrent caries or defective restorations are detected, the patient will undergo appropriate treatment, following the main in-office workflow as previously described. Following the completion of treatment, the nurse will provide oral health education to the patient again, such as guidance on plaque control and sugar intake recommendations based on dietary counseling results, to reinforce caries self-management skills. For special populations, including children, pregnant women, and the elderly, tailored educational materials previously developed, such as customized oral health brochures, can also be used for targeted instruction.

#### 5.2.4. Establish a Distinctive “Enabling System for Diversified Nurse Training”

Within the current Chinese healthcare system, the role of dental nurses is primarily focused on chairside assistance, supplemented by routine duties in health education and material management. Nevertheless, extended responsibilities—such as caries risk assessment, professional fluoride application, pit and fissure sealing, dietary counseling, psychological support, and AI-assisted screening—are not yet governed by a standardized regulatory framework.

Achieving seamless coordination across pre-treatment, intraoperative, and follow-up care requires enhancing dental nurses’ professional competence and clinical autonomy. Thus, a stratified training system is essential to develop oral health managers capable of life-cycle care, ensuring a sustainable talent base for advancing oral healthcare in China. Some potential future directions include the following:

Clinical prevention experts: Proficient in caries risk assessment (CAMBRA), various fluoride applications (gel, varnish, foam), pit and fissure sealants, non-invasive treatment techniques, and oral hygiene guidance skills [57].

Patient educators and communicators: Master effective communication and education methods tailored to different age groups, cultural backgrounds, and health literacy levels.

Basic Nutrition Advisor: With professional nutrition training, they can interpret diet diaries, identify high-caries-inducing dietary patterns, provide basic healthy diet recommendations (limiting sugar intake, choosing artificial sweeteners, dietary fiber), and identify complex cases that require referral to a professional nutritionist [58].

Psychological Supporter: Master the skills for identifying dental anxiety/claustrophobia, apply relaxation techniques (deep breathing guidance), cognitive behavioral therapy (CBT) fundamentals, provide emotional support, and identify patients with severe anxiety who require referral to a psychologist.

Chronic disease managers: Understand the connection between systemic diseases (such as diabetes) and dental caries, and guide patients in self-managing their oral and overall health.

Project Coordination and Community Expansion: Possess the ability to plan, coordinate, implement and evaluate community outreach projects.

Training methods: Systematic on-the-job training, specialized certification courses, interdisciplinary learning, participation in quality improvement projects.

## 6. Limitation and Expectation

Nevertheless, as this article is primarily a narrative review, direct evidence for dentist–nurse integrated caries management in China remains limited, and future studies are needed to evaluate clinical outcomes, patient satisfaction, cost-effectiveness, recall adherence, and caries reduction. Meanwhile, the participation of nurses in dental care management may still encounter real-world barriers including nurse workload, staffing levels, training needs, costs, reimbursement, patient adherence, electronic health record integration, legal liability, and setting-specific differences (tertiary hospitals, community clinics, rural facilities). The following aspects may be prioritized to further strengthen the Integrated Dentist–Nurse Collaborative Care Model for caries management: policy support to expand insurance coverage for nurse-led preventive services [59]; standardization through specialized training and certification programs for nursing staff in caries management [60]; technological enhancement via AI-assisted assessment and remote monitoring to improve diagnostic and collaborative accuracy [61]; and interdisciplinary collaboration integrating nutritionists and psychological counselors into a comprehensive intervention network [59].

## 7. Conclusions

With the increasing incidence of dental caries, the traditional fragmented diagnosis and treatment model has proven inadequate to address the increasingly complex public health challenges. Consequently, the Integrated Dentist–Nurse Collaborative Care Model may serve as a practical approach for caries management. Through task-shifting, this model delegates standardized, repeatable, and preventive tasks to professionally trained nursing teams, which has the potential to improve the healthcare system’s service capacity and efficiency. As a chronic disease, the best treatment strategy for dental caries lies in long-term management and control rather than passive restoration. The integrated care model may establish a closed-loop management process of “screening, assessment, prevention, treatment, and follow-up,” providing continuous and personalized guidance to transform patients’ health behaviors and thus achieve a shift from a “treatment-centered” to a “health-oriented” approach.

## Figures and Tables

**Table 1:** Comparison of work content of dental care personnel in different countries.

Events	Work Content	America		UK		Japan	China
		[19,20,21,22,23,24]		[16,25]		[26,27,28]	[29,30]
		Dental Assistant	Dental Hygienist	Dental Nurse	Dental Hygienist	Dental Hygienist	Dental Nurse
Pre-assessment	Arrange the patients to the appropriate dentists based on their different conditions and lead them to the treatment chairs.	●	●	●	●	●	●
	Record the general information of the patient and the dental treatment records.	●	●	●	●	●	●
	Evaluate the patient’s oral condition, formulate an oral care plan, and implement the plan.		○		✓		
Cooperate with therapeutic procedures	Pass instruments, prepare materials, conduct four-handed operations.	●	●	●	●	●	●
	Assist the doctor in conducting emergency rescue.	✓	✓	✓	✓	✓	●
Direct treatment and nursing procedures	Conduct dental X-ray photography.	✓	✓	●	✓		●
	Relieve patients’ fear and enhance their comfort.	●	●	●	●	●	●
	Prepare and mix dental materials.	●	●	●	●	●	●
	Apply preventive materials such as fluoride and sealants to the teeth.	✓	✓	✓	✓	✓	○
	Remove calculus and plaque from the teeth.		✓		✓	✓	○
	Oral anesthesia.		✓		✓	○	
Oral health education	Regularly conduct publicity on oral healthcare knowledge.	●	●	✓	✓	✓	●
	Give oral healthcare guidance.	●	●	✓	✓	✓	●
Clinical management work	Control management of cross-infection in the clinic, disinfection equipment.	✓	✓	✓	✓	✓	✓
	Ensure the storage, maintenance and preservation of medical devices and materials.	●	●	●	●	●	●

✓ = statutory scope of practice; ● = common clinical practice; ○ = proposed authorized role (discussed in policy proposals or pilot programs, not yet statutory or routine). Sources include references, government websites, and association websites. Laws vary among U.S. states; data are based on Washington, D.C.

## Data Availability

No new data were created or analyzed in this study. Data sharing is not applicable to this article.

## References

[1] World Health Organization (2022). Global Oral Health Status Report: Towards Universal Health Coverage for Oral Health by 2030. https://www.who.int/publications/i/item/9789240061484.

[2] Roberts N.L.S., Mountjoy-Venning W.C., Anjomshoa M., Banoub J.A.M., Yasin Y.J., GBD 2017 Disease and Injury Incidence and Prevalence Collaborators (2019). Global, regional, and national incidence, prevalence, and years lived with disability for 354 diseases and injuries for 195 countries and territories, 1990–2017: A systematic analysis for the Global Burden of Disease Study. Lancet.

[3] National Committee for Oral Health (2018). The Fourth National Epidemiological Survey of Oral Health.

[4] Featherstone J.D.B., Crystal Y.O., Alston P., Chaffee B.W., Doméjean S., Rechmann P., Zhan L., Ramos G.F. (2021). Evidence-Based Caries Management for All Ages-Practical Guidelines. Front. Oral Health.

[5] Cheng L., Zhang L., Yue L., Ling J., Fan M., Yang D., Huang Z., Niu Y., Liu J., Zhao J. (2022). Expert consensus on dental caries management. Int. J. Oral Sci..

[6] Zhou X.D., Cheng L., Zheng L.W. (2018). Strategies of caries management in whole life cycle. Chin. J. Stomatol..

[7] Zhang F., Xie J.J., Chutinan S., Riedy C.A. (2025). Preferred communication techniques by student-providers and patients during caries management in a teaching practice: A quality improvement study. BMC Med. Educ..

[8] Hurlbutt M., Young D.A. (2014). A best practices approach to caries management. J. Evid.-Based Dent. Pract..

[9] Dolce M.C., Barrow J., Jivraj A., Pham D., Da Silva J.D. (2020). Interprofessional value-based health care: Nurse practitioner-dentist model. J. Public Health Dent..

[10] Keboa M., Beaudin A., Cyr J., Decoste J., Power F., Hovey R., LaFrance L., Ouellet D., Wiseman M., Macdonald M.E. (2019). Dentistry and nursing working together to improve oral health care in a long-term care facility. Geriatr. Nurs..

[11] Jassim A., Rodriguez J.M., Kalsi H., Vidal-Nicholson A., Salaver S. (2025). Improving patient care through development of dental nurse specialist roles. Br. Dent. J..

[12] American Dental Association (2026). Introduction of Dental Assistant. https://www.ada.org/resources/careers/career-pathways/dental-assistant.

[13] American Dental Association (2026). Introduction of Dental Hygienist. https://www.ada.org/resources/careers/career-pathways/dental-hygienist.

[14] Morgan M. (2021). Tooth and Veil: The Life and Times of the New Zealand Dental Nurse. Health Hist..

[15] Thompson D.R. (2006). Nursing in Hong Kong: Issues and challenges. Nurs. Sci. Quart..

[16] General Dental Council of the United Kingdom (2023). Dental Nurses Scope of Practice Guidance. https://www.gdc-uk.org/standards-guidance/standards-and-guidance/scope-of-practice.

[17] Ministry of Health, Labour, and Welfare of Japan The Law of Dental Hygienist. https://www.mhlw.go.jp/content/10804000/001363496.pdf#3#1.

[18] Johannsen A., Malmqvist S., Graça S., Assunção V., Albuquerque T., Luis H. (2019). The Dental Hygienists in Sweden and Portugal: A Comparative Study. J. Int. Soc. Prev. Community Dent..

[19] Duque A.D., Malheiros Z., Stewart B., Romanelli H.J. (2020). Strategies for the prevention of periodontal disease and its impact on general health in Latin America. Section III: Prevention. Braz. Oral Res..

[20] Dickinson C., Beatty C.F., Marshall D. (2012). A pilot study: Are dental hygienists in Texas ready for the elderly population explosion?. Int. J. Dent. Hyg..

[21] America Dental Hygiene Association (2013). Dental Hygiene Practice Act Overview: Permitted Functions and Supervision Levels by State.

[22] Inukai J., Sakurai M., Nakagaki H., Matsui K., Matsuda H., Tamura K., Danielsen B., Rowbotham J., Kosaka T. (2012). Comparison of clinical practice education in dental hygiene schools in eight countries. Int. Dent. J..

[23] Gadbury-Amyot C.C., Simmer-Beck M.L., Lynch A., Rowley L.J. (2023). Dental hygiene and direct access to care: Past and present. Int. J. Dent. Hyg..

[24] American Dental Hygienists’ Association (2023). Dental Hygiene Practice Act Overview: Permitted Functions and Supervision Levels by State. https://www.adha.org/wp-content/uploads/2023/05/ADHA-Practice-Act-Overview-5-2023.pdf.

[25] (2025). The Official Government Website for Northern Ireland. Dental Nurse Careers. https://www.nidirect.gov.uk/articles/dental-nurse.

[26] Takahashi Y., Hikiji H., Nishihara T. (2020). A preliminary study of the scope of practice of dental hygienists and oral health providers in Asia. J. Oral Sci..

[27] Muroga R., Tsuruta J., Morio I. (2015). Disparity in perception of the working condition of dental hygienists between dentists and dental hygiene students in Japan. Int. J. Dent. Hyg..

[28] Ministry of Health, Labour and Welfare (2025). Training Program for Implementing Infiltration Anesthesia by Dental Hygienists. https://www.mhlw.go.jp/content/10804000/001521305.pdf.

[29] National Health Commission of the People’s Republic of China (2008). Nurses Regulations. https://www.nhc.gov.cn/fzs/c100048/201808/f10825832c6e4c259af327f055becb3a.shtml.

[30] Yuecen L., Hong R., Huifen W. (2016). Analysis on the status quo of dental clinical nurses’ working scope at home and abroad. Int. J. Nurs..

[31] Cheng L., Zhou X.D. (2021). Clinical assessment of caries prevention and management. Chin. J. Stomatol..

[32] Hillebrecht A.-L., Waterkotte R., Ludwig E., Barbe G. (2024). Integrating risks for oral diseases into Structured Information Collection: A practice development project. Pflege.

[33] White R. (2000). Nurse assessment of oral health: A review of practice and education. Br. J. Nurs..

[34] Boczko F., McKeon S., Sturkie D. (2009). Long-Term Care and Oral Health Knowledge. J. Am. Med. Dir. Assoc..

[35] Daniel B.T., Damato K.L., Johnson J. (2004). Educational issues in oral care. Semin. Oncol. Nurs..

[36] Yoon M.N., Ickert C., Slaughter S.E., Lengyel C., Carrier N., Keller H. (2018). Oral health status of long-term care residents in Canada: Results of a national cross-sectional study. Gerodontology.

[37] Sirsch E., Ludwig E., Mueller K., Blumenberg P., Nitschke I., Buescher A. (2022). Promotion of oral health in nursing-An interprofessional expert standard. Z. Gerontol. Geriatr..

[38] Waldman H.B., Perlman S.P. (2012). Ensuring oral health for older individuals with intellectual and developmental disabilities. J. Clin. Nurs..

[39] Nie E., Jiang R., Islam R., Li X., Yu J. (2024). Evaluation of caries risk assessment practices among dental practitioners in Guangzhou, China: A cross-sectional study. Front. Oral Health.

[40] Munteanu A., Holban A.-M., Pauna M.-R., Imre M., Farcasiu A.-T., Farcasiu C. (2022). Review of Professionally Applied Fluorides for Preventing Dental Caries in Children and Adolescents. Appl. Sci..

[41] Yin T., Sun H., Tang R., Li Q., Zheng J., Feng Y., Wang L. (2024). Impact Assessment of the “4+1 Nursing Operation Mode” on Enhancing the Efficacy of Alveolar Surgery Diagnosis and Treatment. J. Craniofac. Surg..

[42] Gillam D.G., Yusuf H. (2019). Brief Motivational Interviewing in Dental Practice. Dent. J..

[43] Farje-Gallardo C.A., Salazar O.P., Coronel-Zubiate F.T. (2025). Innovative learning in dental education: Integrating narrative and 3D industrial design for teaching caries health disease processes. BMC Oral Health.

[44] Yu O.Y., Lam W.Y.-H., Wong A.W.-Y., Duangthip D., Chu C.-H. (2021). Nonrestorative Management of Dental Caries. Dent. J..

[45] King S., Church L.A., O’Hagan E., Candelaria D., Pawar A., Cooper A., Chen R., Gibson A. (2025). Developing a codesigned text message-based digital oral health education resource (TOOTH). Digit. Health.

[46] Starr J.R., Ruff R.R., Palmisano J., Goodson J.M., Bukhari O.M., Niederman R. (2021). Longitudinal caries prevalence in a comprehensive, multicomponent, school-based prevention program. J. Am. Dent. Assoc..

[47] Liu Z., Li J., Wang S., Liu W., Wang W., Lin H., Pang L. (2026). A Multimodal Model for Caries Screening Using Intraoral Images and Questionnaires. Int. Dent. J..

[48] Perelman S.C., Ansari T., Featherstone J.D.B., Roykh B. (2026). Shifting the Paradigm: CAMBRA Adoption and Medical-Dental Integration Leveraging an Electronic Health Record. J. Dent. Educ..

[49] Featherstone J.D.B., Chaffee B.W. (2018). The Evidence for Caries Management by Risk Assessment (CAMBRA). Adv. Dent. Res..

[50] Research Services and Scientific Information, ADA Library & Archives (2011). Caries Risk Assessment Form (Age 0–6) & (Age > 6). https://www.ada.org/resources/ada-library/oral-health-topics/caries-risk-assessment-and-management.

[51] American Academy of Pediatric Dentistry (2016). Guideline on Caries-risk Assessment and Management for Infants, Children, and Adolescents. Pediatr. Dent..

[52] Bratthall D., Petersson G.H. (2005). Cariogram a multifactorial risk assessment model for a multifactorial disease. Community Dent. Oral Epidemiol..

[53] Tang Y.J., Dong X., Li R., Zhang X. (2025). Construction and practice of a stratified training program based on nursing clinical ladder model and Benner novice to expert theory in novice dental nurses. Chin. J. Integr. Nurs..

[54] Griffin S.O., Jones K., Gray S.K., Malvitz D.M., Gooch B.F. (2008). Exploring four-handed delivery and retention of resin-based sealants. J. Am. Dent. Assoc..

[55] Holmes D.C., Squire L.J., Arneson S.K., Doering J.V. (2009). Comparison of Student Productivity in Four-Handed Clinic and Regular Unassisted Clinic. J. Dent. Educ..

[56] Bayne S.C., Schmalz G. (2005). Reprinting the classic article on USPHS evaluation methods for measuring the clinical research performance of restorative materials. Clin. Oral Investig..

[57] Baik A., Alamoudi N., El-Housseiny A., Altuwirqi A. (2021). Fluoride Varnishes for Preventing Occlusal Dental Caries: A Review. Dent. J..

[58] Sonmez M., Akben M., Gocebe B. (2023). Evaluation of oral hygiene behaviors and teeth condition of students in oral and dental health nursing course. Acta Sci.-Health Sci..

[59] Giacaman R.A., Fernandez C.E., Munoz-Sandoval C., Leon S., Garcia-Manriquez N., Echeverria C., Valdes S., Castro R.J., Gambetta-Tessini K. (2022). Understanding dental caries as a non-communicable and behavioral disease: Management implications. Front. Oral Health.

[60] Sadura Z., Hanks S., Tredwin C., McColl E. (2021). The dental therapist’s role in a ‘shared care’ approach to optimise clinical outcomes. Br. Dent. J..

[61] Liang Y., Li D., Deng D., Chu C.H., Mei M.L., Li Y., Yu N., He J., Cheng L. (2025). AI-Driven Dental Caries Management Strategies: From Clinical Practice to Professional Education and Public Self Care. Int. Dent. J..

